# Adaptation and Utilization of a Postmarket Evaluation Model for Digital Contact Tracing Mobile Health Tools in the United States: Observational Cross-sectional Study

**DOI:** 10.2196/38633

**Published:** 2023-03-22

**Authors:** Kevin E Cevasco, Amira A Roess

**Affiliations:** 1 College of Health and Human Services George Mason University Fairfax, VA United States

**Keywords:** COVID-19, contact tracing, postmarketing, mobile apps, public health, digital, interventions, tool, adoption, effectiveness, prevention, application, transmission

## Abstract

**Background:**

Case investigation and contact tracing are core public health activities used to interrupt disease transmission. These activities are traditionally conducted manually. During periods of high COVID-19 incidence, US health departments were unable to scale up case management staff to deliver effective and timely contact-tracing services. In response, digital contact tracing (DCT) apps for mobile phones were introduced to automate these activities. DCT apps detect when other DCT users are close enough to transmit COVID-19 and enable alerts to notify users of potential disease exposure. These apps were deployed quickly during the pandemic without an opportunity to conduct experiments to determine effectiveness. However, it is unclear whether these apps can effectively supplement understaffed manual contact tracers.

**Objective:**

The aims of this study were to (1) evaluate the effectiveness of COVID-19 DCT apps deployed in the United States during the COVID-19 pandemic and (2) determine if there is sufficient DCT adoption and interest in adoption to meet a minimum population use rate to be effective (56%). To assess uptake, interest and safe use covariates were derived from evaluating DCTs using the American Psychological Association App Evaluation Model (AEM) framework.

**Methods:**

We analyzed data from a nationally representative survey of US adults about their COVID-19–related behaviors and experiences. Survey respondents were divided into three segments: those who adopted a DCT app, those who are interested but did not adopt, and those not interested. Descriptive statistics were used to characterize factors of the three groups. Multivariable logistic regression models were used to analyze the characteristics of segments adopting and interested in DCT apps against AEM framework covariates.

**Results:**

An insufficient percentage of the population adopted or was interested in DCTs to achieve our minimum national target effectiveness rate (56%). A total of 17.4% (n=490) of the study population reported adopting a DCT app, 24.7% (n=697) reported interest, and 58.0% (n=1637) were not interested. Younger, high-income, and uninsured individuals were more likely to adopt a DCT app. In contrast, people in fair to poor health were interested in DCT apps but did not adopt them. App adoption was positively associated with visiting friends and family outside the home (odds ratio [OR] 1.63, 95% CI 1.28-2.09), not wearing masks (OR 0.52, 95% CI 0.38-0.71), and adopters thinking they have or had COVID-19 (OR 1.60, 95% CI 1.21-2.12).

**Conclusions:**

Overall, a small percentage of the population adopted DCT apps. These apps may not be effective in protecting adopters’ friends and family from their maskless contacts outside the home given low adoption rates. The public health community should account for safe use behavioral factors in future public health contact-tracing app design. The AEM framework was useful in developing a study design to evaluate DCT effectiveness and safety.

## Introduction

### Background

During periods of high COVID-19 incidence, US health departments were unable to scale up case management staff to deliver effective and timely contact-tracing services [1]. This is expected considering that prior to the COVID-19 outbreak, US health departments were grossly understaffed and needed a minimum of 80,000 more full-time equivalent positions to provide adequate infrastructure and a minimum package of public health services [2].

Digital contact tracing (DCT) apps are intended to supplement health department case managers by automatically and more efficiently estimating the proximity and duration of an individual’s exposure to patients diagnosed with COVID-19. A model developed to predict the impact of conventional and mobile DCT apps on the SARS-CoV-2 pandemic estimated that the disease transmission rate could be reduced by adding either contact tracers or effective widely used DCT apps (eg, Bluetooth, GPS) [3]. DCTs reduce disease spread by alerting the user if they have been near an infected person who is also using DCT, followed by user isolation and testing for infection. However, a key DCT effectiveness assumption is that there are sufficient people adopting the app to signal disease exposure. Ongoing monitoring of population uptake is necessary to address concerns that users are relying on DCT apps that have not met minimum population-level app adoption rates [4,5].

### Health Technology Assessments

Health technology assessments (HTAs) of public health applications are uncommon and challenged by lack of data, conflicting stakeholder priorities, and methodological issues [6]. The surge in COVID-19 cases in the United States in the summer of 2021 (ie, the “third wave”) driven by the SARS-CoV-2 Delta variant marks an urgent time to perform an assessment of DCT apps to inform future public health contact-tracing programs and app design [7]. By 2020, an estimated 2.6 billion people globally were expected to use mobile health (mHealth) apps, generating an estimated US $31 billion in revenue [8].

Despite the large market of mHealth apps, there is no US regulatory authority in place to evaluate if DCT apps are safe and beneficial [9]. Section 522 of the Federal Food, Drug, and Cosmetic Act provides the Food and Drug Administration (FDA) with the authority to require manufacturers to conduct postmarket surveillance to address important public health questions on the effectiveness and safety of a health device [10]. However, the FDA generally does not regulate products that are intended solely to track locations or contacts associated with public health surveillance [11]. Public health DCT developers could voluntarily implement HTAs using the FDA Software Precertification Program; however, the FDA guidance does not provide a framework to conduct the postmarket evaluations [12].

There are a wide variety of frameworks available for evaluating health applications, but they may not be suitable for HTAs [13,14]. The clinical applications marketplace conducts HTAs, but they have not been frequently performed in the public health market [6]. This study adapted the American Psychological Association (APA) App Evaluation Model (AEM) hierarchical rating system because it was developed by harmonizing application evaluation questions from 45 frameworks [15]. The AEM provides a comprehensive evaluation framework for clinicians and patients to identify high-quality mHealth apps. The framework may work well at a population level with hierarchical layers to explain adoption decisions [16,17].

DCT only functions when all people near each other are using the app. Individuals install DCT apps on their own mobile devices, and these apps must be adopted by a sufficient percentage of a population to be effective [16,17]. In this study, we set the adoption effectiveness rate at 56% of the national population [18]. We define our DCT adoption as the percentage of the population using a DCT plus those that are interested in using a DCT but have not yet adopted it. Our assumption is that public health campaigns could convert many of the interested users into adopters through public health communication campaigns.

Following public health safety guidance while using DCT is important, and the AEM framework provides health and technology evaluation elements to evaluate functional use. For example, DCT requires users to voluntarily report their positive COVID-19 test results to public health authorities; therefore, user behavior is part of DCT functionality. After DCT adoption, users should not cease their social distancing behaviors in a way that increases disease exposures.

DCT represents a new technology with a short research history, and the AEM provides a structure for measure selection [19]. The AEM has five categories for evaluation: Access and Background, Privacy and Safety, Clinical Foundation, Usability, and Data Integration Toward a Therapeutic Goal. The AEM provides a way to critically assess mHealth and does not include a minimum or maximum score to be considered “good” or “useful” [15,20].

### Objective

The main objective of this study was to evaluate COVID-19 DCT apps deployed in the United States during the COVID-19 pandemic under the hypothesis that DCT requires a minimal level of uptake by a population to be effective and DCT users should follow social distancing policies. Accordingly, the specific study aims were as follows.

Aim 1: Determine if there is a sufficient percentage of people using or interested in DCT to reach the minimum critical mass (56%) [18].

Aim 2: Assess the effectiveness and safety of DCT apps using the APA AEM hierarchical rating system [16].

The AEM is a conceptual framework for developing key study measures and was used to select variables. The measures derived from the framework assess adoption rate characteristics and behaviors necessary for the safe use of DCT apps.

## Methods

### Data Source

We approximated a nationally representative sample of US adults (aged 18+ years), with a stratified nonprobability sample to survey 3853 online panel participants between December 22, 2020, and January 2, 2021. Respondents were recruited by Climate Nexus Polling, using several market research panels as described elsewhere [21]. Participants were recruited using stratified sampling methods [21]. Compensation for participants depended on the specific market research panel and respondents’ preferences (eg, cash, gift cards, reward points) and was valued below US $4.00. Quotas were set to match the US Census Bureau’s Voting and Registration Supplement to the Current Population Survey parameters for age, gender, race, educational attainment, census region, and Hispanic ethnicity. Sampling weights were used to account for small deviations from the preselected census parameters. The participation rate of the survey was 68.5% (qualified respondents that completed the survey). The 95% credibility interval for this survey was ±1.7%. The survey data are provided in Multimedia Appendix 1.

DCT apps are new technology and there were no established and validated survey instruments to measure their use; therefore, Climate Nexus Polling developed DCT app survey questions based on a previous Pew Research survey on digital privacy [22].

### Key Study Measures Aligned to the APA AEM

#### Model Variable Selection

**Figure 1:**
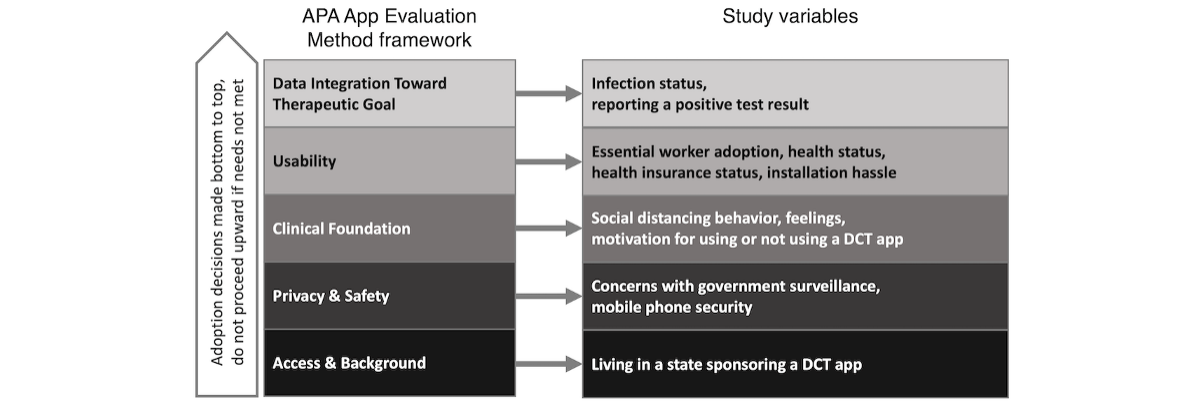
App Evaluation Model (AEM) with study variables. APA: American Psychological Association; DCT: digital contact tracing.

Demographic measures were based on a literature review to assess if app adoption applied to a diverse range of people [23,24]. The following background for each category links the AEM to the study’s DCT postmarket evaluation. The AEM framework was used to select variables a priori from data available in the survey (Figure 1). The detailed process is described in Multimedia Appendix 2.

#### Access and Background

This category is concerned with the transparency of app governance and ensuring that the benefits of mHealth apps are available to a diverse range of people, regardless of background. The US DCT apps are based on mobile phone Bluetooth proximity tracking using the Google/Apple Exposure Notification (GAEN) platform [25], and are cosponsored by US state government public health organizations [25]. Adoption of DCT apps in the United States is voluntary, and varies by demographics and behavior [23]. Furthermore, DCT app adoption may be subject to health equity issues since mHealth adoption is generally associated with younger, educated populations [23,26].

The “state of residence” measure was used to demonstrate the role of a person’s state government in driving local adoption [25,27,28].

#### Privacy and Safety

There is an expectation of confidentiality by DCT users, and privacy policies should ensure that COVID-19 exposure proximity measurement is accurate. Concern over privacy and data breaches is a genuine concern. Experts collaborated to develop secure decentralized contract-tracing platform architectures [29]; however, some GAEN implementations may have exposed user data. A class action lawsuit was filed in April of 2021 against Google, claiming that their COVID-19 contact-tracing platform exposed unwitting Android adopters’ sensitive personal information [30].

Concerns over government surveillance and mobile device hacking measures were selected from the survey [23,28,30].

#### Clinical Foundation

There is no agreed-upon definition of effectiveness for measuring the clinical foundation of DCT. In January 2021, the National Institute of Standards and Technology held a workshop to discuss successes and challenges associated with implementing proximity detection technologies [31]. For public health, effectiveness is defined as reducing transmission and quicker notification of exposure. From an adopter perspective, effectiveness lies in addressing privacy concerns and finding a value proposition. From a technology perspective, effectiveness is how well the DCT apps measure proximity and if they can function at current adoption rates. A systematic review of DCT also found this lack of definition for effectiveness [18].

Also of potential concern is the “risk compensation” theory, which suggests that individuals will increase risky social distancing behavior after a COVID-19 public health intervention is implemented [32,33]. Researchers are concerned that DCT app adoption could create a false sense of security and safety, leading to behavior that increases infections [34]. Additionally, the personality of the adopter may be an issue because the extroversion personality type is associated with lower compliance with social distancing [35].

Clinical foundation measures were selected to inform future work on the definition of DCT public health effectiveness. Measures were selected to assess social distancing behavior (mask, visiting friends/family, attending religious services) [23,35]. Psychological distress that could impact adoption and measures were included to assess a user’s worry about COVID-19 [36]. Measures on motivations for adoption and interest were selected from the survey [23,24].

#### Usability

Grounded in the principle of autonomy, an mHealth app should enable the user to take an educated and active role in their health decisions. For example, DCT apps could help essential workers take an active role in managing their exposures. However, a German study showed that there was not greater uptake of DCT apps by people working during the early pandemic [23]. In addition, the readability of DCT apps is higher than US reading levels and potentially less accessible for the general population [27]. There is also little known about DCT use by people in good versus poor health. Studies show that older people with chronic diseases adopt self-care and vitals mHealth apps [26]; however, their chronic disease management is a very different purpose than tracking infectious disease exposure.

Usability is especially important for populations with high exposure risk or at high risk of poor outcomes. Measures were selected for essential worker, health, and health insurance status [23,24].

#### Data Integration Toward a Therapeutic Goal

This category is related to the principle of shared decision-making through appropriate information sharing with public health departments and health care systems. Some DCT apps do offer adopters links to testing resources, local health department resources, and quarantine social support [27]. In the US GAEN DCT systems, adopters are solely in control of reporting their positive test results through the app; therefore, designers have limited ability to automate the contact-tracing reporting data path. Unfortunately, a Morbidity and Mortality Weekly Report found that 35% to 48% of individuals with COVID-19 did not report any of their contacts to public health department contact tracers [37]. DCT apps provide anonymous COVID-19–positive lab test reporting capability, but there is no evidence showing that DCT adoption improves the likelihood of reporting.

User infection status and sharing a positive test result measures assess if apps ultimately provide exposure data to public health authorities [24,37].

### Statistical Analysis

Market segments of the survey population were divided into three categories: (1) the “adopted” category includes the population self-declaring use of a DCT app; (2) the “interested-not-adopted” category includes the population self-declaring interest in, but not using a DCT app; and (3) the “not interested” category describes the remaining population. All three categories were included in the descriptive statistics table using frequencies and survey weighted percentages. Only the adopted and interested-not-adopted (“interested”) categories were included in the statistical analysis because these populations would be targets for uptake communications campaigns. Logistic regression models were applied to the adopted and interested-not-adopted segments to compare against the key measures. Regression results for the two models are presented with unweighted percentages and adjusted odds ratios (ORs) with 95% CIs. Both model results and key measures are presented in tables with variables organized by AEM categories. Health care workers (n=1029) were excluded from the analysis because health systems had contact-tracing protocols in place before the COVID-19 pandemic to meet health care–acquired infections regulations. Health care workers may answer survey questions inconsistently because they follow workplace infection contact-tracing protocols that are not applicable to the general public. Multicollinearity of adopted and interested-not-adopted models were checked through correlation analysis. Descriptive and statistical analyses were conducted using STATA version 16.1 (StataCorp, College Station, TX).

To mitigate the risk of model overfitting given the large number of measures, variables were selected a priori through a literature review and allocated to the APA framework as described in the Key Measures section above and Multimedia Appendix 2. By using measures with statistical significance from other data sets, we reduced the likelihood that our results were based on overfitting in our survey sample. Results were also analyzed for overfit risk by setting a minimum measure subsample frequency threshold (n>15) [38].

### Ethical Considerations

This project was considered exempt for review by the George Mason University Institutional Review Board (1684418-3).

## Results

### Descriptive Analysis

Out of 3853 respondents, 2824 were included in the analysis, with 1029 excluded because they self-identified as health care workers. Among the total sample of 2824 people, 490 (17.4%) reported using a DCT app, 697 (24.7%) reported interest, and 1637 (58.0%) were not interested (Table 1). All measures exceed the minimum frequency threshold for assessing the risk of overfitting, with most of the measures having over 100 survey respondents.

Older age groups adopted the apps less frequently, where the ≥65-year-old group are at higher risk of poor COVID-19 outcomes but represent a lower rate of adoption (10.2%). Those not interested in adopting the app were disproportionately concerned with government surveillance postepidemic (43.2%).

**Table 1:** Frequencies of survey population and survey segments.

Survey segment			Study population (N=2824), n (wt%^a^)	Adopted (n=490), n (%)	Interested-not-adopted (n=697), n (%)	Not interested (n=1637), n (%)
Access and background: State of residence offers a DCT^b^ app (yes)			1534 (55.3)	310 (63.3)	376 (54.0)	848 (51.8)
**Security and privacy**						
	Do not use because of concerns with government surveillance postepidemic		N/A^c^	N/A	236 (33.9)	707 (43.2)
	Do not use because my phone might get hacked		N/A	N/A	155 (22.2)	405 (24.7)
**Clinical foundation**						
	**Social distancing**					
		Visit friends and family weekly (yes)	1321 (47.6)	302 (61.6)	286 (43.0)	733 (45.1)
		Worn a mask in public to help protect yourself or others from getting sick? (yes)	2409 (84.5)	367 (74.9)	611 (87.7)	1431 (87.4)
		Attends religious services (yes)	1309 (46.5)	285 (58.2)	333 (47.8)	691 (42.2)
	**Feelings**					
		Worried about losing your home (yes)	784 (28.5)	212 (43.3)	215 (30.9)	357 (21.8)
		Feel afraid (yes)	893 (31.9)	204 (41.6)	271 (38.9)	418 (25.5)
		Feel lonely (yes)	812 (28.6)	202 (41.2)	230 (33.0)	380 (23.2)
	**Motivation for use**					
		Use to protect family and friends	N/A	279 (56.9)	430 (61.7)	N/A
		Use to help stop the pandemic/reduce deaths in older American adults	N/A	229 (46.7)	326 (46.8)	N/A
		Use as a responsibility to my community	N/A	219 (44.7)	320 (45.9)	N/A
		Use to let me know my risk of infection/help peace of mind/help me stay healthy	N/A	268 (54.7)	432 (62.0)	N/A
		Not use because it would make me feel more anxious	N/A	N/A	227 (32.6)	552 (33.7)
		Not use because I would not benefit/I won’t be infected	N/A	N/A	122 (17.5)	446 (27.2)
**Usability**						
	Essential worker		664 (24.5)	157 (32.0)	173 (24.8)	334 (20.4)
	**Health status**					
		Very good or excellent	1288 (45.7)	266 (54.3)	317 (45.5)	705 (43.1)
		Good	967 (34.2)	151 (30.8)	226 (32.4)	590 (36.0)
		Fair or poor	569 (20.2)	73 (14.9)	154 (22.1)	342 (20.9)
		Has health insurance (yes)	2421 (85.3)	389 (79.4)	616 (88.4)	1416 (86.5)
	No use because too much hassle to install		N/A	N/A	187 (26.8)	389 (23.8)
**Therapeutic goal**						
	Would you tell your neighbors or friends if you become COVID-19–positive? (yes)		1949 (69.1)	330 (67.4)	502 (72.0)	1117 (68.2)
	Do you think you have already had the coronavirus or currently have it? (yes)		490 (18.2)	140 (28.6)	115 (16.5)	235 (14.4)
**Demographics**						
	**Age (Gallup divisions, years)**					
		18-29	510 (19.0)	155 (31.6)	144 (20.7)	211 (12.9)
		30-49	948 (33.2)	214 (43.7)	283 (40.6)	451 (27.6)
		50-64	686 (24.2)	71 (14.5)	154 (22.1)	461 (28.2)
		65+	680 (23.6)	50 (10.2)	116 (16.6)	514 (31.4)
	**Gender**					
		Female	1491 (51.7)	245 (50.0)	393 (56.4)	853 (52.1)
		Male	1333 (48.3)	245 (50.0)	304 (43.6)	784 (47.9)
	**Race**					
		White	2126 (72.1)	319 (65.1)	506 (72.6)	1301 (79.5)
		Black	322 (10.2)	69 (14.1)	84 (12.1)	169 (10.3)
		Hispanic	232 (14.1)	68 (13.9)	68 (9.8)	96 (5.9)
		Other	144 (3.7)	34 (6.9)	39 (5.6)	71 (4.3)
	**Political party affiliation**					
		Republican	1066 (38.1)	154 (31.4)	205 (29.4)	707 (43.2)
		Democratic	1273 (44.5)	269 (54.9)	383 (55.0)	621 (37.9)
		Independent	485 (17.4)	67 (13.7)	109 (15.6)	309 (18.9)
	**Annual income level (US $)**					
		<50,000	1606 (57.4)	244 (49.8)	366 (52.5)	996 (60.8)
		50,000-99,000	778 (27.4)	137 (28.0)	201 (28.8)	440 (26.9)
		≥100,000	440 (15.2)	109 (22.2)	130 (18.7)	201 (12.3)
	**Population density**					
		Urban	814 (29.5)	185 (37.8)	236 (33.9)	393 (24.0)
		Semiurban	1326 (46.8)	218 (44.5)	322 (46.2)	786 (48.0)
		Rural	684 (23.8)	87 (17.8)	139 (19.9)	458 (28.0)
	**Education**					
		No high school	176 (6.7)	38 (7.8)	44 (6.3)	94 (5.7)
		At least some high school	718 (26.4)	107 (21.8)	147 (21.1)	464 (28.3)
		Some college	1025 (37.7)	158 (32.2)	246 (35.3)	621 (37.9)
		Bachelor’s degree or above	905 (29.3)	187 (38.2)	260 (37.3)	458 (28.0)

^a^wt%: weighted percentage.

^b^DCT: digital contact tracing.

^c^N/A: not applicable.

DCT app adopters more frequently visited friends and family outside the home each week (61.6%) compared to the overall survey rate (47.6%). Those interested in the app visited friends and family outside the home less frequently (43.0%). Adopters also had high rates of concern about losing their home (43.3%), being lonely (41.2%), and feeling afraid (41.6%). Despite having a higher rate of thinking they have or had the coronavirus infection (28.6%), their rate of telling neighbors and friends was similar to that of the base population (67.4%).

There was also a difference in the rate of wearing masks in the overall study population (84.5%) versus a lower rate in the adoption segment (74.9%). Adopters showed a higher rate of very good/excellent health (54.3%) compared to the overall population and other categories.

### Logistic Regression Analysis

There were differences in the frequency and association between the adopted and interested segment populations across all five AEM categories (Table 2). Covariate-adjusted ORs show how the adoption and interest vary by study subject characteristics. The OR is interpreted as the odds of a covariate being the same (OR=1.0) for adopted versus not adopted groups. For example, people who live in a state that offers a DCT adopt the apps 1.47 times more often (OR>1.0) than people in states not offering DCTs. People who wore a mask in public to help protect themselves or others from getting sick adopt DCT apps 0.52 times less often (OR<1.0) than people that do not wear masks.

**Table 2:** Logistic regression results for adopted and interested-not-adopted segments.

Survey segments					Adopted							Interested-not-adopted			
					Respondents (n=2824), %^a^			Adjusted OR^b^ (95% CI) (n=490)			Respondents (n=2334), %^a^				Adjusted OR (95% CI) (n=697)
Access and background: state of residence offers a DCT^c^ app (yes)					*63.3^d^*			*1.47 (1.16-1.86)*			53.9				0.98 (0.81-1.19)
**Security and privacy**															
	Do not use because of concerns with government surveillance postepidemic			N/A^e^			N/A			*25.0*				*0.67 (0.54-0.83)*	
	Do not use because my phone might get hacked			N/A			N/A			*27.7*				*0.78 (0.62-0.99)*	
**Clinical foundation**															
	**Social distancing**														
		Visit friends and family weekly (yes)	*22.9*			*1.63 (1.28-2.09)*			*28.1*				*0.81 (0.66-0.99)*		
		Worn a mask in public to help protect yourself or others from getting sick? (yes)	*15.2*			*0.52 (0.38-0.71)*			29.9				1.05 (0.77-1.44)		
		Attends religious services (Yes)	21.8			1.26 (0.99-1.61)			*32.5*				*1.29 (1.06-1.58)*		
	**Feelings**														
		Worried about losing your home (yes)	*27.0*			*1.52 (1.18-1.95)*			37.6				1.19 (0.94-1.49)		
		Feel afraid (yes)	22.8			1.04 (0.80-1.35)			*39.3*				*1.38 (1.10-1.73)*		
		Feel lonely (yes)	*24.9*			*1.31 (1.00-1.70)*			37.7				1.14 (0.90-1.44)		
	**Motivation for use**														
		Use to protect family and friends	*39.4*			*2.65 (2.00-3.51)*			61.7				—^f^		
		Use to help stop the pandemic/reduce deaths in older American adults	*41.3*			*2.04 (1.52-2.73)*			46.8				—		
		Use as a responsibility to my community	*40.6*			*1.49 (1.10-2.03)*			45.9				—		
		Use to let me know my risk of infection/help peace of mind/help me stay healthy	*38.3*			*2.09 (1.57-2.78)*			62.0				—		
		Do not use because it would make me feel more anxious	N/A			N/A			*29.1*				*0.59 (0.47-0.73)*		
		Do not use because I would not benefit/I won’t be infected	N/A			N/A			21.5				1.14 (0.90-1.44)		
**Usability**															
	Essential worker (yes)			23.6			1.12 (0.87-1.46)			34.1				1.06 (0.84-1.34)	
	**Health status**														
		Very good or excellent	20.7			1			31.0				1		
		Good	15.6			0.93 (0.72-1.22)			27.7				1.10 (0.88-1.38)		
		Fair or poor	12.8			0.73 (0.52-1.03)			*31.0*				*1.42 (1.08-1.85)*		
		Has health insurance (yes)	*16.1*			*0.65 (0.48-0.89)*			30.3				1.24 (0.92-1.68)		
	Do not use because too much hassle to install			N/A			N/A			32.5				0.93 (0.73-1.17)	
**Therapeutic goal**															
	Would you tell your neighbors or friends if you become COVID-19–positive? (yes)			67.4			0.84 (0.65-1.08)			72.0				1.20 (0.97-1.50)	
	Do you think you have already had the coronavirus or currently have it? (yes)			*28.6*			*1.60 (1.21-2.12)*			32.9				0.96 (0.74-1.26)	
**Demographics**															
	**Age (years)**														
		18-29	30.4			1			40.6				1		
		30-49	22.6			0.93 (0.69-1.25)			38.6				0.86 (0.65-1.15)		
		50-64	*10.3*			*0.69 (0.47-1.00)*			*25.0*				*0.47 (0.34-0.64)*		
		65+	*7.4*			*0.63 (0.41-0.98)*			*18.4*				*0.30 (0.21-0.43)*		
	Gender: male			18.4			1.14 (0.90-1.44)			27.9				0.89 (0.73-1.08)	
	**Race**														
		White	15.0			1			28.0				1		
		Black	21.4			1.06 (0.74-1.53)			33.2				0.87 (0.63-1.20)		
		Hispanic	29.3			1.25 (0.86-1.83)			41.5				1.30 (0.90-1.87)		
		Other	23.6			1.45 (0.90-2.34)			35.5				1.01 (0.65-1.57)		
	**Political party affiliation**														
		Republican	14.4			1			22.5				1		
		Democratic	21.1			1.05 (0.80-1.39)			*38.1*				*1.62 (1.29-2.03)*		
		Independent	13.8			0.85 (0.59-1.22)			26.1				0.97 (0.73-1.30)		
	**Annual income level (US $)**														
		<50,000	15.2			1			26.9				1		
		50,000-99,000	17.6			1.10 (0.82-1.47)			31.4				1.13 (0.89-1.43)		
		≥100,000	*24.8*			*1.45 (1.02-2.06)*			*39.3*				*1.42 (1.05-1.91)*		
	**Population density**														
		Urban	22.7			1			37.5				1		
		Semiurban	16.4			0.98 (0.75-1.28)			29.1				0.86 (0.68-1.08)		
		Rural	12.7			0.93 (0.66-1.31)			*23.3*				*0.73 (0.55-0.96)*		
	**Education**														
		No high school	21.6			1			31.9				1		
		Some high school	14.9			1.02 (0.62-1.67)			24.1				0.76 (0.49-1.16)		
		Some college	15.4			0.86 (0.53-1.40)			28.4				0.96 (0.63-1.46)		
		Bachelor’s degree and above	20.7			0.92 (0.55-1.53)			36.2				1.24 (0.80-1.93)		

^a^Frequency is presented as a percentage of adopted or interested-not-adopted against the total population of each variable.

^b^OR: odds ratio.

^c^DCT: digital contact tracing.

^d^Statistically significant associations are in italics.

^e^N/A: not applicable.

^f^Removed from model due to collinearity with being in the interested-not-adopted segment.

Essential worker status was not associated with being in the adoption or interest segments, and education was not a significant factor. Being an adopter was negatively associated with having health insurance (16.1%; OR 0.65, 95% CI 0.48-0.89) and being interested was associated with being in fair to poor health (31.0%; OR 1.42, 95% CI 1.08-1.85). Older age was negatively associated with both adoption and interest. Having an annual income over US $100,000 was associated with both adoption and interest. Political party affiliation was not a statistically significant factor in adoption, although those self-declared as Democratic party members had the highest adoption frequency.

Not adopting a DCT app due to concerns about government surveillance (25.0%; OR 0.65, 95% CI 0.52-0.81) and that their phone will be hacked (27.7%; OR 0.78, 95% CI 0.62-0.99) was negatively associated with interest. Adoption was positively associated with visiting friends and family (22.9%; OR 1.63, 95% CI 1.28-2.09), whereas interest was negatively associated with visits (28.1%; OR 0.81, 95% CI 0.66-0.99). Religious services attendance was associated with interest (32.5%; OR 1.29, 95% CI 1.06-1.58). Adopters tended to express feeling lonely (24.9%; OR 1.31, 95% CI 1.00-1.70), while being interested was positively associated with feeling afraid (39.3%; OR 1.38, 95% CI 1.10-1.73). Motivation measures showed the highest frequencies in the adoption (38.3%-41.3%) and interest segments.

Adopters were more likely to think they have or had the coronavirus (28.6%; OR 1.60, 95% CI 1.21-2.12). Adoption was not positively associated with telling friends and neighbors about a positive COVID-19 lab result.

## Discussion

### Principal Findings

Overall, each layer of the hierarchical AEM framework shows barriers to the ability of DCT to effectively and safely supplement understaffed manual contact tracers and protect the public. With respect to aim 1, we found that a small percentage of the eligible population reported adoption of the app (17.4%), which is lower than estimated adoption rates in the United Kingdom (28%) [39] and New Zealand (31%) [40]. Programs to encourage adoption among those interested in a DCT app (24.7%) could increase adoption, but still fall well below the minimum target threshold of adoption. The not-interested segment is large enough (58.0%) to prevent DCT apps from reaching the population adoption minimum. Low adoption and interest rates are problematic because in order for DCT apps to be effective, high population uptake is needed alongside other control measures [18]. With respect to aim 2, inconsistent associations across prior DCT studies may be due to variable selection differences, which shows the benefit of using the AEM framework to develop study measures a priori [19]. Our results show that DCT apps may not be clinically effective in protecting adopters’ friends and family from their frequent maskless contact outside the home given low adoption rates and DCT technology false-negative detection issues. Adopter and interested segments were positively associated with motivations to protect friends and family, community, and the older population; however, this study found several areas of concern regarding beneficence.

Essential workers are at greater risk of exposure as part of their employment, but they adopted DCT apps at a low rate (23.6%). Essential workers were not associated with being part of either the adoption or interest segment, and other studies also found that they had lower intent to adopt and report positive COVID-19 lab tests [24]. Future DCT app design should consider that infection risk varies across subpopulations, and DCT solution design should focus on adoption with higher-risk subpopulations. A study on DCT app user sentiment suggested that DCT apps are largely passive in nature, and could be improved by adding features such as proactive reminder notifications and links to detailed disease-spread information [30].

The US public health department sponsorship of DCT apps was considered a positive attribute in the AEM Access and Background category; however, in the not-interested segment, this was a negative attribute due to the association with distrust of government surveillance. Other studies indicated concerns about privacy that varied by political affiliation [28], whereas concerns about postpandemic surveillance and hacking were not associated with adoption hesitancy in the interested segment. Resolving this conundrum requires further study of the not-interested segment.

DCT apps are available for free on Google Play and Apple Store, and the adoption rate is higher in the 26 US states sponsoring apps [25]. Early studies before DCTs were deployed could only survey people about their intent to adopt [24], and our findings show differences in adoption versus interest subpopulations across the AEM categories. The influence of age, gender, race, and income was found to be inconsistent across studies [24] and variation in culture is suggested to play a role [23]. Our results support previous findings that DCT adoption is associated with a younger population, but did not support the association with well-educated populations [23,26].

Safe use of DCT apps after adoption is a concern. The high volume of social visits warrants further investigation into the influence of risk compensation behavior and an extraversion personality trait. The COVID-19 pandemic has resulted in ongoing related psychological stress accompanying DCT exposure monitoring, reporting, quarantine, and isolation [41]. Adoption and interest are associated with mental health stressors, with worries of losing a home and feelings of loneliness. More research is needed to determine if there are causal links with loneliness, extroversion personality type, DCT adoption, and poor social distancing compliance [35]. Our study suggests that DCT apps should not be considered as passive public health infection reporting tools because of the psychological and behavioral associations [36]. Our findings support a prior recommendation to investigate DCT adoption psychological impacts [36].

Neither adoption nor interest was associated with telling neighbors or friends about testing positive for COVID-19. This is concerning considering that adoption was associated with adopters thinking they had been infected with the coronavirus. DCTs may not be an effective addition to manual contact tracing given that they are not associated with improving upon the rate of infected people sharing contacts with public health contact tracers. Lessons from other stigmatized diseases such as HIV may offer DCT designers insight to encourage the sharing of exposure information to slow transmission [42]. Findings from an HIV mHealth prevention and testing study recommended using evidence-based methods for app development, including links to geospatial prevention information and the ability to access self-testing kits [43]. There is a clinical trial underway for an HIV mHealth app that may produce useful information to DCT developers [44].

### Limitations

Adoption and interest were measured by self-described use at the time of the survey and did not include questions about duration of use. The survey was national and did not ask the respondents which of the DCT apps they considered when answering survey questions, although most states use the same GAEN proximity detection platform. The study results occasionally varied from those reported by international DCT deployments, which suggests that local culture can play a role in adoption. The data are representative of the US adult population but not of all app adopters, and we were able to identify important differences between adopters and nonadopters. Importantly, this study is the first to create a postmarket assessment approach for DCTs, and points to the need to prioritize development of multidisciplinary teams at each step of the development, implementation, and evaluation continuum.

This study did not assess the political party control of DCT sponsoring states, which controls if a DCT is offered and how energetically it is marketed. An individual’s political party affiliation was not associated with adoption, but political party in control of a regional government is likely a different factor than individual user political affiliation.

### Conclusions

With low adoption rates nationally, the DCT apps may only be useful as part of pandemic management programs in smaller, more targeted populations such as company employees, university employees and students, or health system workers and clients.

The AEM highlighted key clinical foundation issues that should be addressed in exposure contact-tracing system design. For example, DCT app adoption is associated with frequent visits with friends and family members outside the home, not wearing masks, not having health insurance, feeling lonely, and adopters thinking they have/had the coronavirus. Designing for behavioral risks requires public health and technology organizations to codevelop multidisciplinary definitions of DCT effectiveness along with measurement methods.

The APA AEM framework combined with observational data analysis is suited to assess end-user mobile app experience and can help with future study measure selection. This postmarket beneficence and safety evaluation approach using the AEM and observational data could be applied to other COVID-19 pandemic public health applications that manage disease exposure, promote personal health tracking, monitor health, and raise awareness [45].

## Appendix

Multimedia Appendix 1 Survey data used in a postmarket evaluation model for digital contract tracing (January 2021).

Multimedia Appendix 2 App evaluation model variable selection process.

## Abbreviations

AEM: App Evaluation Model
APA: American Psychological Association
DCT: digital contact tracing
FDA: Food and Drug Administration
GAEN: Google/Apple exposure notification
HTA: health technology assessment
mHealth: mobile health
OR: odds ratio

## Data Availability

A subset of survey data that were used in this study and the data dictionary are available in Multimedia Appendix 1.
