# Supplementary material for: Adaptation and Utilization of a Postmarket Evaluation Model for Digital Contact Tracing Mobile Health Tools in the United States: Observational Cross-sectional Study
Source: JMIR Public Health Surveill. 2023 Mar 22;9:e38633. doi: 10.2196/38633 (PMC10036112; doi:10.2196/38633)
Supplement: Multimedia Appendix 2 [file publichealth_v9i1e38633_app2.docx]

# Multimedia Appendix 2

# AEM Screening questions mapped to measures and survey questions

This study applied the American Psychological Association (APA) App Evaluation Model (AEM) framework using a health technology assessment, postmarket evaluation perspective. The literature review identified areas of public health research interest within the AEM framework. Table 3 shows AEM screening questions aligned with measures and survey questions.

**Adopt, Interest Groups**

Adopt and Interest groups were created using these DCT specific survey questions that asked subjects if they have heard of, used, and were interested in DCT applications.

Survey Questions:

Have you heard of contact tracing apps that use movement tracking data to notify individuals of possible exposures to COVID-19 positive individuals? For example, the apps that Google and Apple have developed, or COVID-Wise.

- Are you currently using such an app?
- Are you interested in using such an app?

**AEM Question Exclusion Criteria**

The AEM model was designed to help APA members, patients, and providers become familiar with important information to considered when picking an app. However, not all AEM App Evaluation Model questions are appropriate for this postmarket assessment and GAEN application architecture. Therefore, the evaluation excluded and grouped AEM questions for the following four reasons (marked as N/A):

1. The GAEN architecture and public health application sponsorship governance limits how application developers can design notification exposure features. AEM questions prescribed by or inappropriate to the GAEN architecture and public health governance were excluded.
2. Public health organizations posted the DCTs independently in the Apple and Google stores; therefore, AEM questions involving individual app updates and app related purchases were excluded.
3. Some questions were grouped to measure uptake through the FDA User Experience Analytics (UXA) evaluation concept ^1^.
4. Questions about effectiveness of the DCTs have been excluded because the NIST workshop^2^ showed there is no clear public health nor technical definition of effectiveness.

Table 3: AEM evaluation questions mapped to measures

| **Comprehensive App Evaluation Model Questions** | **Map of study measures and survey questions to AEM** |
| --- | --- |
| **Step 1: Access and Background** | |
| 1. Does the app identify ownership? | A state sponsorship measure was created to link the survey subject’s location to residency within states that sponsor applications. The residency variable was generated based on survey subject living in a state listed in the MIT COVID Tracing Tracker^a^. The measure accounts for the role of state public health departments driving local adoption and interest. |
| 1. Does the app identify funding sources and conflicts of interest? |  |
| 1. Does the app come from a trusted source? |  |
| 1. Does it claim to be medical? | N/A |
| 1. Are there additional or hidden costs? | N/A |
| 1. Does the app work offline? | N/A |
| 1. On which platforms/operating systems does it work? | N/A |
| 1. Does it work on a desktop computer? | N/A |
| 1. Does the app work with accessibility features of the iPhone/android? | N/A |
| 1. Is it accessible for those with impaired vision or other disabilities? | N/A |
| 1. Has the app been updated in the last 180 days? | N/A |
| **Step 2: Privacy and Security** | |
| 1. Is there a transparent privacy policy that is clear and accessible before use? | User response measures for privacy and security were selected to align with the FDA Software Precertification recommendation to assess end user experience. The measures represent the user’s security and privacy perspective of the GAEN architecture DCTs.  Survey Questions: “Why are you NOT using the app or why would you NOT be interested in using such an app?   - Concerns with government surveillance post-epidemic - My phone might get hacked |
| 1. Does the app declare data use and purpose? |  |
| 1. Does the app describe use of PHI? |  |
| 1. Deidentified vs. anonymous? |  |
| 1. Can you opt out of data collection or delete data? |  |
| 1. Are data maintained in the device or on the web? |  |
| 1. Does the app explain security systems used? |  |
| 1. Does the app collect, use, and/or transmit sensitive data? If yes, does it claim to do so securely? |  |
| 1. What third parties does the app share data with? |  |
| 1. If appropriate, is the app equipped to respond to potential harms or safety concerns? |  |
| **Step 3: Clinical Foundation** | |
| 1. Does the app appear to do what it claims to do? | Measures were selected to help the public health community understand DCT application adoption associations with social distancing behavior. Measures were included to assess compliance with several non-pharmaceutical interventions: frequency of visits with people outside of home, use of masks to limit transmission, and frequency of attending religious services.  Survey Questions: How many times, if at all, have you done the following over the past 7 days?   - Gotten together with family or friends who don't live with you? - Worn a mask in public to help protect yourself or others from getting sick? - Aside from weddings and funerals, how often do you attend religious services? |
| 1. Is the app content correct, well-written, and relevant? | N/A |
| 1. What are the relevant sources or references supporting the app use cases? | The motivation measures were included to assess end user’s postmarket reasons for using and not using DCTs. The measures can help public health DCT development teams improve use cases by understanding DCT user’s personal motivations.  Survey Questions: Why are you using the app or why would you be interested in using such an app?   - To protect family and friends - It might help stop the pandemic/reduce deaths in elderly Americans - A responsibility to my community - It would let me know my risk of infection/help peace of mind/help me stay healthy   Survey Questions: Why are you NOT using the app or why would you NOT be interested in using such an app?   - I would not benefit/I won't be infected - It would make me feel more anxious |
| 1. Is there evidence of specific benefit from academic institutions, publications, end user feedback, or research studies? | N/A |
| 1. Is there evidence of effectiveness/efficacy? | N/A |
| 1. Was there an attempt to validate app usability and feasibility? | N/A |
| 1. Does the app have a clinical/recovery foundation relevant to your intended use? | Measures for loneliness, worry, and fear were included to help the public health community understand the mental health status of DCT users.  Survey Questions: How strongly do you feel each of the following emotions when you think about the coronavirus?   - Lonely - Afraid   How worried are you about each of the following as a result of the spread of the coronavirus?   - Losing your home |
| **Step 4: Usability** |  |
| 1. What are the main engagement styles of the app? | N/A |
| 1. Do the app and its features align with your needs and priorities? | The survey did not include questions on individual interactions with DCT applications; therefore, this study focused on the public health concern with the DCT applications use by populations at high risk. User experience measures were assigned for high exposure risk and health vulnerability.  Essential worker measures are included due to their high rates of COVID-19 exposure and frequent public interaction during outbreaks and periods and non-pharmaceutical intervention policies that restrict movement.  Survey Question:   - Are you an essential worker required to work in close contact with others (e.g. retail salesperson, cashier, bus/taxi driver, firefighter, police officer, security guard, custodial staff, restaurant staff, maid, childcare worker, construction worker, other)?   Measures for people in poor health and without health insurance are included due to risk of severe outcomes if infected.  Survey Questions:   - Now we would like to ask you a few questions about your health. In general, is your health excellent, very good, good, fair, or poor? - Are you, yourself, now covered by any form of health insurance or health plan or do you not have health insurance at this time? |
| 1. Is it customizable? | N/A |
| 1. Does the app clearly define functional scope? | N/A |
| 1. Does the app seem easy to use? | Why are you NOT using the app or why would you NOT be interested in using such an app?   - Too much hassle to install |
| **Step 5: Data Integration towards Therapeutic Goal** | |
| 1. Do you own your data? | N/A |
| 1. Can data be easily shared and interpreted in a way that's consistent with the stated purpose of the app? | N/A |
| 1. Can the app share data with EMR and other data tools (apple Healthkit, FitBit)? | N/A |
| 1. Is the app for individual use or to be used in collaboration with a provider? | Adopters exposed to COVID-19 must give permission in their DCT application to allow public health departments to access their personal exposures. A measure on a user’s intent to share positive infection status is included to assess associations with infection reporting intent.  Survey Question:   - Would you tell your neighbors or friends if you become COVID positive? |
| 1. If intended to be used with a provider, does the app have the ability to export or transfer data? |  |
| 1. Does the app lead to any positive behavior change or skill acquisition? | A COVID diagnosis measure is included to assess if the user has been infected and the association between DCT application adoption/intent and infection.  Survey Question:   - Have you been diagnosed with the coronavirus by a medical professional? |
| 1. Does the app improve therapeutic alliance between patient and provider? |  |

*N/A – No study measure associated with this screening question.
^a^ The Covid Tracing Tracker [25]*

**Endnote**

^1^ Food and Drug Administration. Developing a Software Precertification Program [12]

^2^ Workshop Report: Challenges for Digital Proximity Detection in Pandemics [31]
